# Sexual well-being in old age: A systematic review of the literature

**DOI:** 10.1192/j.eurpsy.2023.544

**Published:** 2023-07-19

**Authors:** S. von Humboldt, J. A. Ribeiro-Gonçalves, G. Low, I. Leal

**Affiliations:** ^1^William James Center for Research, ISPA - Instituto Universitário, Lisbon, Portugal; ^2^Faculty of Nursing, University of Alberta, Edmonton, AB, Canada

## Abstract

**Introduction:**

We conducted a systematic review of the qualitative literature on the sexuality and sexual health of older adults.

**Objectives:**

The aim is to address which topics have been researched and the quality of research within this field.

**Methods:**

All stages of this review were carried out peer-to-peer in order to guarantee minimized bias. The Cochrane Database, Psy-EBSCO, MEDLINE, Psy-Redalyc, Scielo, Web of Science and Google Scholar were searched and 32 studies met inclusion criteria. The majority had not been reviewed in earlier review articles. A total of 95,478 references were screened and 27 studies were included in this review. The studies involved 3044 participants across seven countries, most being women (approximately 83%).

**Results:**

We identified a wide variety of factors that can determine SWB of older adults, such as perceived health, sexual health, demonstrations of love; non-sexual joint activities; overall well-being and quality of life; partner support; positive self-image; being independent and active; the strength of spiritual beliefs, and patriarchal roles upheld by upbringings conveying that women’s role is to provide men with sexual pleasure.

**Conclusions:**

Methodological issues related to sampling procedures, such as purposive sampling through the older samples and limited generalisability due to the homogeneity of participants. Additionally, there was a widespread lack of non-heterosexual control groups. However, most studies used appropriate measures and acknowledged inherent limitations. There is a lack of research with the older population, those with significant health needs, those outside the Western countries, and those with additional characteristics associated with discrimination.

**Disclosure of Interest:**

None Declared

